# Protein Lactylation in Liver Disease: A Comprehensive Review

**DOI:** 10.1017/erm.2026.10036

**Published:** 2026-02-09

**Authors:** Sipu Wang, Jie Zhang, Chao Sun

**Affiliations:** 1 Department of Gastroenterology and Hepatology, Tianjin Medical University General Hospital, Tianjin, China; 2 Department of Gastroenterology, The First Affiliated Hospital of Xi’an Medical University, Xi’an, China

**Keywords:** lactate, lactylation, liver disease, pharmacology, post-translational modification, treatment

## Abstract

**Background:**

Lactate, generated through glycolysis, plays a dual role as both a metabolic substrate and a signalling molecule, influencing cellular functions in pathophysiological scenarios. Protein lactylation, a recently identified form of post-translational modification mediated by lactate, has garnered significant and increasing attention. Globally, hepatic disorders pose a significant public health burden, frequently involving disruptions in glucose metabolism and consequent lactate buildup.

**Methods:**

This comprehensive review examines the discovery, regulatory mechanisms and pathogenic roles of lactylation in diverse liver disorders, while critically evaluating emerging lactylation-targeted therapeutics to guide future translational research.

**Results:**

Lactylation modifications play a pivotal role in various pathophysiological processes, including hepatic inflammation, liver fibrosis, ischaemic injury, tumour growth and metastasis.

**Conclusions:**

Modulation of lactylation pathways, coupled with pharmacological control of lactate synthesis and shuttling, emerges as a strategic approach to liver disease therapeutics.

## Introduction

Proteins are integral to the execution of a wide array of cellular and physiological processes in different organisms, encompassing metabolism, catalysis, signalling and movement (Ref. 1). The functional diversity of proteins is significantly enhanced through alternative splicing and post-translational modifications (PTMs) of transcripts. It is estimated that selective splicing alone produces approximately 100,000 human protein isoforms, while the combined effects of splicing and PTMs generate proteomes numbering at least in the tens of millions (Refs 2, 3). The stability of proteins, regulated by PTM, is key to maintaining homeostasis and preventing disease (Ref. 4). PTMs include glycosylation, ubiquitination, SUMOylation, acetylation, phosphorylation, palmitoylation and lactylation, among others, and play a critical role in influencing nearly all facets of cellular biology and pathology. Notably, lactylation represents a recently identified PTM within the past half-decade. Despite the nascent stage of research on lactylation, it has already demonstrated significant medicinal potentials requiring intense scrutiny.

The seminal identification of lactylation, a new class pertinent to PTM, has been achieved by Zhang and colleagues in 2019 via detection of lysine residue mass shifts in proteolytic peptides consistent with lactyl group adduction (Ref. 5). They unravelled that lactate is converted into lactyl-CoA, which is then transferred to the lysine residues of histones by lactyltransferase, thereby establishing the process of histone lactylation. Alternatively, Gaffney et al. found a non-enzymatic lactylation process that operates independently of particular enzymes (Ref. 6). Findings from their study demonstrated that lactoyl-glutathione, a glycolysis intermediate, can transfer lactyl groups to lysine residues on proteins. This process is intricately regulated by glyoxalase II, a key enzyme in the glyoxalase system. Later, Wan and team verified that lactylation occurs not only on histones but is also widely found as a PTM on non-histone proteins (Ref. 7). As research into lactylation has progressed, its roles across a variety of diseases, including immune disorders, cancer, cardiovascular diseases and neurological conditions, have been increasingly uncovered and elucidated (Refs 8–11).

Annually, liver-related illnesses affect about 150 million people worldwide and lead to nearly 2 million fatalities, highlighting a significant global health concern (Ref. 12). Among these, non-alcoholic fatty liver disease (NAFLD) alone affects about 25% of the global adult population (Refs 13–15). In the body, the liver primarily catabolizes lactate, transforming it into pyruvate, and then produces glycogen and glucose through the process of gluconeogenesis (Ref. 16). The liver typically demonstrates the highest rate of lactate removal in the body (Ref. 17). Impaired liver function results in dysregulation of lactate metabolism. In those suffering from chronic hepatopathy, the removal of lactate is significantly diminished, which is responsible for its accumulation (Ref. 18). Thus, liver damage severely impairs its capability to metabolize and eliminate lactate, giving rise to increased lactate levels in response to diverse pathological conditions. Simultaneously, various hepatocyte types exhibit elevated glycolytic activity due to the liver’s high levels of lactate (Ref. 19). Collectively, the purpose of this review is to summarize the molecular mechanisms and clinical relevance pertinent to protein lactylation in the context of liver disease. To better understand this issue, the mechanistic actions and biological functions of lactylation modification will also be summarised and discussed in depth.

## Regulation of lactylation

### Lactate production and clearance

Identified in 1780, lactate was historically conceptualised as a hypoxia-induced metabolic waste product causally linked to pathological outcomes in oxygen-deprived tissues (Ref. 20). Within the cytosol, glucose undergoes a series of catalytic reactions to form pyruvate, which is subsequently converted to lactate through lactate dehydrogenase A (LDHA) (Ref. 21). Early in the 20th century, Warburg and colleagues discovered that tumour cells preferentially generate lactate through glycolysis even under oxygen-sufficient conditions following glucose uptake; this metabolic shift, known as the Warburg effect, contributes to tumour proliferation and invasion (Ref. 22). Beyond glycolysis, glutamine metabolism serves as another significant source of lactate in cancer cells. Under the regulation of transcription factor c-Myc, glutamine is transported into the cytosol via amino acid transporters system ASC amino acid transporter 2 (ASCT2) and system N amino acid transporter 2 (SN2). Subsequently, glutaminase (GLS/GLS2) catalyses the deamination of glutamine to form glutamate. There are two main pathways for the further metabolism of glutamate: 1) via glutamate dehydrogenase or 2) via transaminases, such as gluta mate oxaloacetate transaminase and glutamate pyruvate transaminase and phosphoserine aminotransferase, to yield α-ketoglutarate (α-KG). The resulting α-KG enters the tricarboxylic acid (TCA) cycle. Within the TCA cycle, the carbon skeleton derived from glutamine is ultimately converted to oxaloacetate, then transformed into malate and shuttled back to the cytosol. Cytosolic malate undergoes a reverse reductive reaction catalysed by malic enzyme 1 to produce NADPH and pyruvate. The generated NADPH participates in fatty acid and cholesterol biosynthesis as well as the antioxidant defence system, while pyruvate serves as the precursor for lactate production. Therefore, the glutamine metabolic pathway not only supplies carbon sources for lactate synthesis but also constitutes a crucial alternative route for lactate generation in cancer cells (Ref. 23). Excessive bodily lactate accumulation can lead to lactic acidosis; therefore, organisms actively clear lactate from tissues through distinct metabolic pathways (Ref. 24). The primary method of lactate clearance involves its oxidation to pyruvate, which is then catalysed by pyruvate dehydrogenase to form acetyl-CoA. Acetyl-CoA subsequently enters the TCA cycle, ultimately generating CO₂ and water in addition to providing energy (Ref. 25). Beyond serving as a glycolytic end-product, lactate may be diverted towards glucose resynthesis through gluconeogenic pathways or, alternatively, be channelled to lactoyl-CoA, thereby supplying the donor substrate for the enzymatic addition of lactyl groups towards non-histone and histone targets (Ref. 26).

### Lactate transport

Monocarboxylate transporters (MCTs), belonging to the solute carrier 16 (SLC16) family, are important proteins responsible for lactate transport across membranes (Ref. 27). Among the 14 identified MCT isoforms, MCT1–4 is widely expressed in various tissues. Under physiological conditions, the coordinated action of these isoforms facilitates the bidirectional shuttling of lactate between glycolytically active cells and oxidative cells, playing a crucial role in maintaining lactate homeostasis across different tissues. In normal tissues, MCT1, characterised by its high affinity for lactate, serves as the central transporter for lactate homeostasis, whose activity is strictly modulated in alignment with the transmembrane lactate concentration gradient. On the other hand, in cells where intracellular lactate levels remain high, such as tumour cells, the primary pathway for lactate export is the low-affinity transporter MCT4. The MCT-dictated lactate transport mechanism relies on a proton gradient: free protons first bind to the transporter, followed by lactate binding which initiates a conformational change in the transport complex. This ultimately releases lactate to the opposite side of the membrane and elicits proton dissociation subsequently. Following the release of protons, MCT reverts to its initial conformation, primed for the next catalytic cycle. MCT1 dysfunction (dysregulated expression or inactivating mutations) is associated with several disorders, including but not limited to symptomatic deficiency in lactate transport, familial hyperinsulinemic hypoglycaemia 7 and monocarboxylate transporter 1 deficiency (Ref. 17). Notably, MCT1, MCT2 and MCT4 exhibit marked overexpression in various cancers. The lactate exchange facilitated by these transporters orchestrates metabolic coupling between tumour cells in addition to forging a synergistic metabolic network connecting glycolytic tumour cells to oxidative tumour cells, in consequence driving tumourigenesis, progression and adaptive metabolic reprogramming (Refs 28, 29).

### Lactylation regulation enzymes

Regarding the process of PTMs towards proteins, three key players are often involved: ‘erasers’ (enzymes that remove modifications), ‘writers’ (enzymes that catalyse covalent addition of specific groups) and ‘readers’ (effector proteins that bind modified proteins) (Ref. 30). The primary function of lactylation ‘writers’ is to add lactyl groups to lysine residues. The first enzyme reported as a ‘writer’ mediating histone lactylation is p300 (Ref. 5). Studies using genetic knockdown or deletion experiments have confirmed that silencing p300 significantly reduces the lactylation level of the non-histone protein high mobility group box 1 (HMGB1) (Refs 31, 32). Other enzymes reported to possess lactylation writer activity incorporate p300/ CREB-binding protein (p300/CBP), general control non-depressible 5 (GCN5), tat interacting protein (TIP60), alanyl-tRNA synthetase 1-2 (AARS1–2), lysine acetyltransferase 8 (KAT8), YiaC and MYST-A (Ref. 19). In contrast to ‘writers’, histone deacetylases (HDACs) function as ‘erasers’ to remove lactylation. Research conducted by Moreno et al. demonstrated that HDAC1 and HDAC3 specifically remove lactyl-lysine modifications from histones (Ref. 33). HDAC1–3 is therefore considered the most effective lactylation ‘erasers’ in vitro. Additionally, sirtuin 1–3(SIRT1–3) exhibit substantial lactylation erasure activity (Refs 34, 35). Progress in defining lactylation ‘reader’ proteins has been relatively limited. Because lysine acetylation and lactylation are chemically similar, it has been suggested that some well-known acetyl-lysine readers may also recognize lactyl-lysine marks (Ref. 36). Hu et al. showed that the bromodomain-containing protein BRG1 binds H3K18la during the early stage of induced pluripotent stem cell reprogramming and is involved in chromatin remodelling (Ref. 37). Nunez et al. reported TRIM33 as a putative reader of H3K18la (Ref. 38). Zhai et al. further identified DPF2 as a reader of H3K14la and proposed that the DPF2–H3K14la interaction affects chromatin remodelling and gene expression in cancer, partly by promoting programmes that support cell survival and tumour formation (Ref. 39).

## Lactate metabolism and functions

Energy Metabolism: Lactate, a key metabolic intermediate, can be reversibly converted to pyruvate under the catalysis of lactate dehydrogenase B (LDHB). This pyruvate molecule not only serves as a precursor for gluconeogenesis to synthesize glucose, thereby providing energy for the organism (Ref. 40) but also contributes widely to cellular energy metabolism. Notably, lactate triggers the release of intracellular Mg^2+^ from the endoplasmic reticulum. This Mg^2+^ transport mechanism is tightly coordinated with core metabolic feedback loops and mitochondrial bioenergetic functions, collectively harnessing mitochondrial Mg^2+^ homeostasis (Ref. 41).

Regulation of fatty acid metabolism: The synthesis of fatty acids within cells is powerfully driven by lactate, which enhances the acetyl-CoA pool and the catalytic function of acetyl-CoA carboxylase. In terms of immunomodulation, lactate triggers the upregulation of the lactate transporter solute carrier family (SLC) 5A12 in CD4^+^ T cells. This SLC5A12-dependent lactate uptake establishes a positive feedback loop, further reinforcing the fatty acid synthesis pathway (Ref. 17). Concurrently, lactate exerts dual regulatory effects on fatty acid oxidation (FAO). For instance, under inflammatory stress, acetyl-CoA generated from FAO can promote glycolytic flux via non-enzymatic acetylation, ultimately giving rise to increased lactate production (Ref. 42).

**Figure 1.**
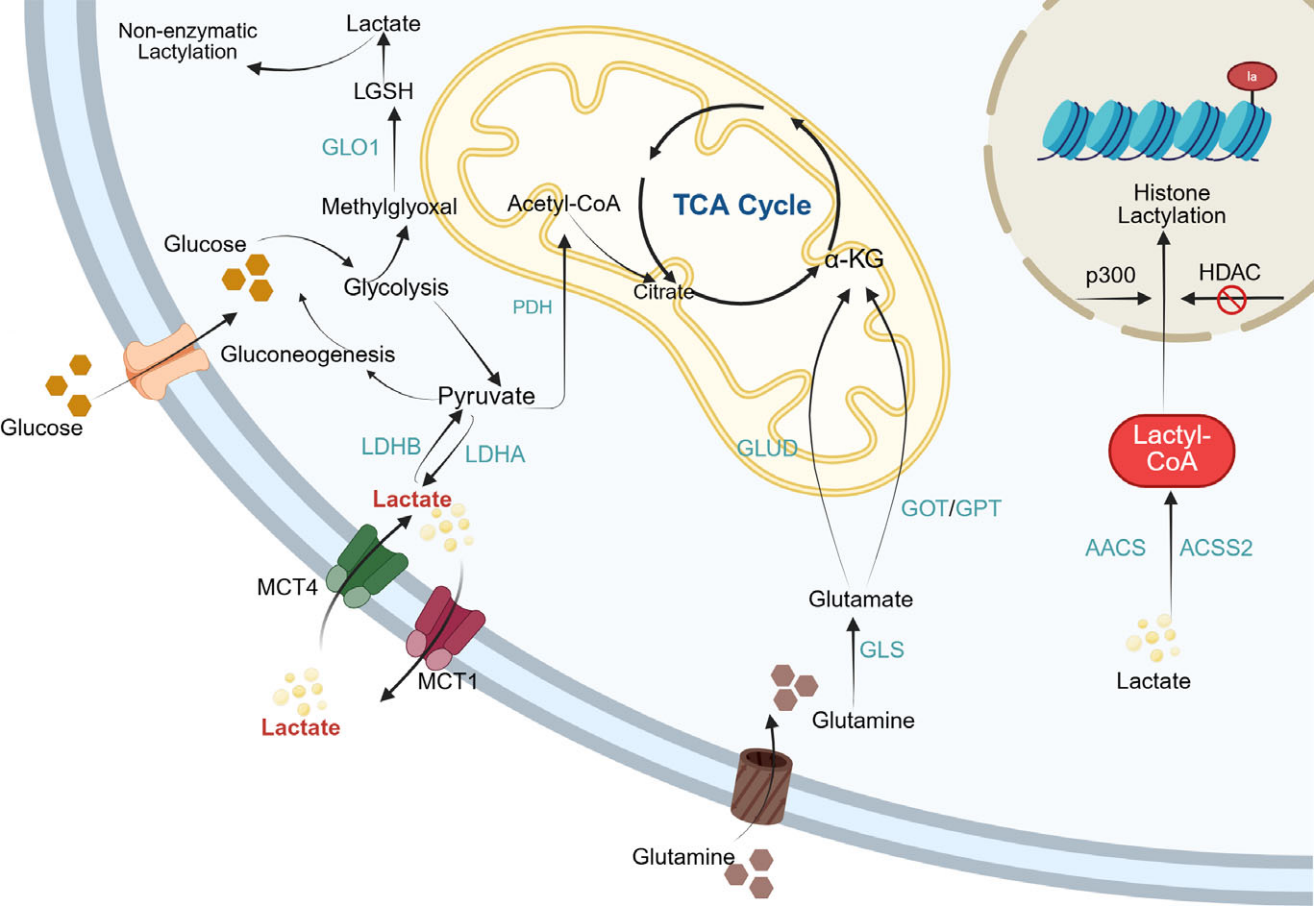
Lactate metabolism and lactylation in cells. Within the cytoplasm, lactate enters cells via MCTs and is generated through both glycolysis and glutaminolysis. Intracellular lactate metabolism proceeds through two distinct pathways. In one pathway, lactate is oxidised to pyruvate, which subsequently enters the mitochondria and is metabolised via the TCA cycle. Alternatively, lactate can be converted to lactoyl-CoA, which then participates in the lactylation of both histones and non-histone proteins. MCT: Monocarboxylate transporter; LDHA: lactate dehydrogenase A; LDHB: lactate dehydrogenase B; PDH: pyruvate dehydrogenase; GLUD: glutamate dehydrogenase; GOT: glutamateoxaloacetate transaminase; GPT: glutamate-pyruvate transaminase; GLS: glutaminase; AACS: acetoacetyl-CoA synthetase; ACSS2: acetyl-CoA synthetase 2; HDAC: histone deacetylases; LGSH: lactoylglutathione.

Lactate functions as a key redox signalling molecule and metabolic energy source, driving the oxidative metabolism of multiple tissues and playing a crucial role in sustaining redox homeostasis and tissue stability (Ref. 43). The underlying mechanism involves the capture of electrons released during substrate oxidation by oxidised nicotinamide adenine dinucleotide (NAD^+^ or NADP^+^), forming reduced coenzymes (NADH or NADPH). Subsequently, these reduced coenzymes are re-oxidised, releasing electrons through the mitochondrial respiratory chain or lactate fermentation pathways, thereby fine-tuning intracellular redox balance (Ref. 44). For clarity, Figure 1 illustrates the overall framework linking lactate generation, intracellular transport and lactylation-related enzymatic reactions, providing a visual summary of the metabolic context described above. The major biological processes mediated by lactate, along with their associated outcomes, are summarised in Table 1.

**Table 1.** Lactate-mediated biological processes and their functional outcomes

Biological process	Mechanistic role of lactate or lactylation	Functional/pathophysiological outcome	References
Energy metabolism	Lactate is reversibly converted to pyruvate by LDHB and promotes Mg^2+^ release from the ER to coordinate mitochondrial activity.	Supports gluconeogenesis and energy homeostasis.	(Refs 51, 52)
Fatty acid metabolism	Lactate increases the acetyl-CoA pool and activates ACC; SLC5A12-mediated lactate uptake enhances fatty acid synthesis.	Links glycolytic flux to lipid metabolism.	(Refs 17, 42)
Glycolysis and glutamine metabolism	LDHA-driven lactate production and glutamine metabolism supply substrates for pyruvate, α-KG, and lactyl-CoA formation.	Integrates carbon flux with metabolic and epigenetic regulation.	(Refs 21–23, 26)
Lactate transport and shuttling	MCT1/2/4 mediate bidirectional lactate exchange between glycolytic and oxidative cells under proton gradient control.	Maintains systemic lactate balance and metabolic coupling.	(Refs 27–29)
Redox homeostasis	Lactate modulates the NADH/NAD^+^ ratio and influences mitochondrial oxidative phosphorylation.	Preserves intracellular redox balance.	(Refs 43, 44)
Immunometabolic regulation	Lactate uptake via SLC5A12 drives fatty acid synthesis and affects T-cell differentiation and macrophage polarisation.	Regulates immune function and inflammation.	(Ref. 17)

## Lactylation in hepatopathy

### Metabolic-associated fatty liver disease

**Figure 2.**
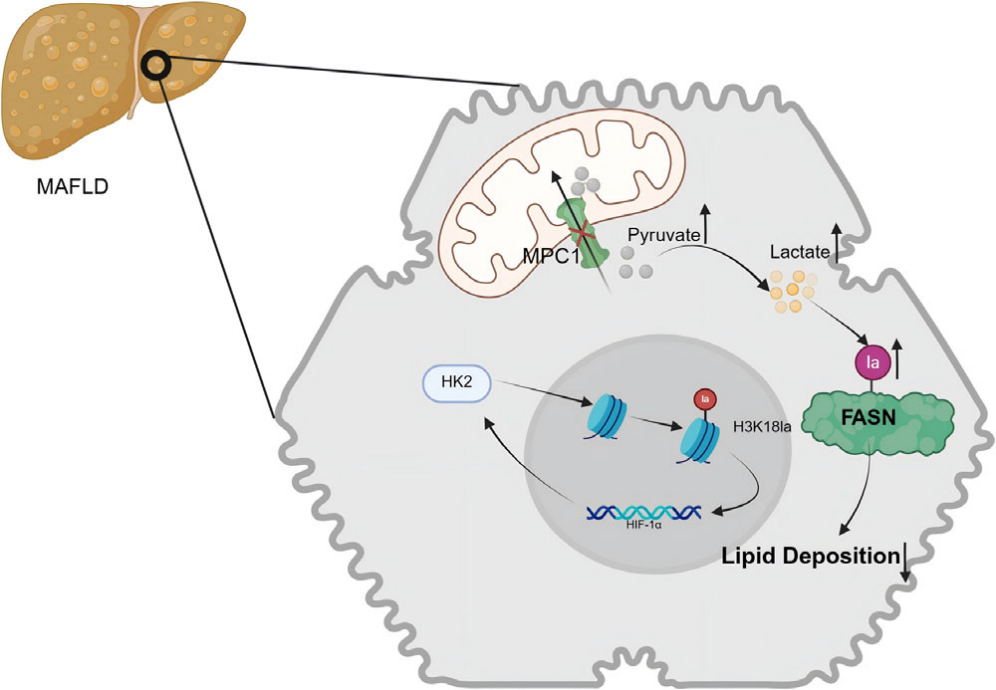
The mechanisms of lactylation in MAFLD. Elevated MPC1 expression positively correlates with hepatic lipid deposition in MAFLD. Knocking down MPC1 increases lactate levels in hepatocytes, facilitating FASN lactylation and thereby mitigating the progression of MAFLD. Moreover, HK2 and H3K18la expression is markedly upregulated and associated with metabolic dysregulation and inflammation, with HIF-1α acting as a key transcriptional regulator. MAFLD: metabolic-associated fatty liver disease; MPC1: mitochondrial pyruvate carrier; FASN: fatty acid synthase; HK2: hexokinase 2.

In 2020, a consensus was reached among 22 countries to rename NAFLD as metabolic-associated fatty liver disease (MAFLD) (Refs 13, 45). In 2024, the American Association for the Study of Liver Diseases termed it metabolic dysfunction-associated steatotic liver disease (MASLD), and this designation later expanded to other regions (Refs 46, 47). MAFLD is recognised as the predominant cause of chronic liver disease globally, with an estimated prevalence of 25% worldwide (Ref. 48). MAFLD is characterised by the presence of hepatic steatosis, as identified through imaging techniques or histopathological evaluation, after excluding alternative secondary aetiologies responsible for hepatic lipid accumulation (Ref. 49). The spectrum of MAFLD encompasses metabolic-associated simple fatty liver (MAFL), metabolic-associated steatohepatitis, and extends to include liver fibrosis, cirrhosis and hepatocellular carcinoma (HCC) (Ref. 50). Mitochondrial pyruvate carrier 1 (MPC1) stands for a pivotal functional carrier protein located on the inner mitochondrial membrane. According to the findings of Gao et al., the expression of MPC1 progressively increases with the severity of fatty degeneration in patients with MAFLD (*n* = 46) (Ref. 51). As illustrated in Figure 2, the loss of MPC1 disrupts mitochondrial pyruvate import, leading to elevated lactate accumulation and enhanced FASN lactylation, which collectively reduce hepatic lipid deposition. Li et al. demonstrated an upregulation of hexokinase 2 (HK2) and H3K18la expression in the liver macrophages of patients and mice with MAFLD (Ref. 52). This upregulation results in increased histone lactylation, which subsequently activates the promoters and transcription of glycolytic genes. The activation of these genes facilitates glycolysis and the M1 polarisation of liver macrophages, culminating in metabolic dysfunction and inflammation within the liver macrophages.

### Liver fibrosis/cirrhosis

**Figure 3.**
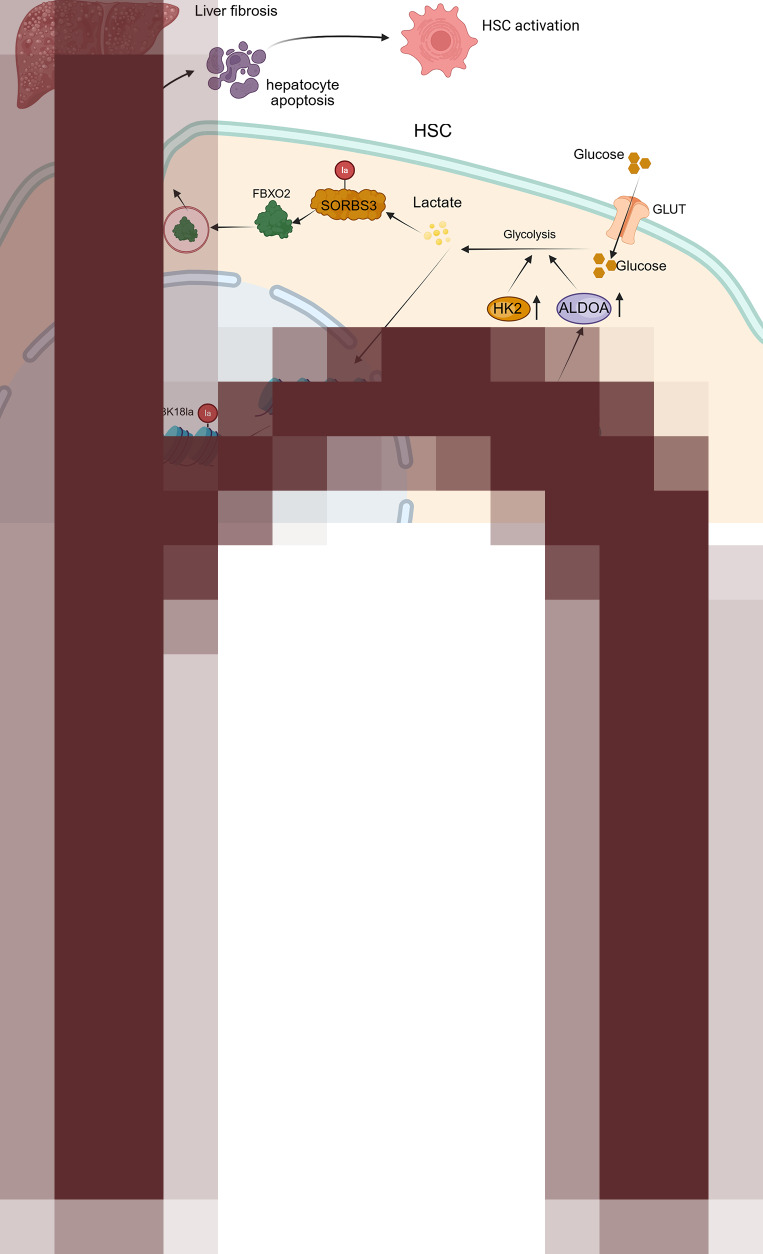
During liver fibrosis, HSCs exhibit enhanced glycolysis and consequent intracellular lactate accumulation. IGF2BP2 augments ALDOA expression via m6A-dependent mRNA stability regulation. ALDOA and HK2, both rate-limiting enzymes in the glycolytic pathway, facilitate glycolysis and lactate production when overexpressed. Lactate-mediated histone lactylation, in turn, promotes the transcription of genes associated with HSCs activation. Furthermore, lactate stimulation elevates the lactylation level of SORBS3, which stimulates the formation of FBXO2-positive small secretory vesicles. Subsequent hepatic uptake of these vesicles contributes to liver injury. HSC: hepatic stellate cell; IGF2BP2: insulin-like growth factor 2 mRNA-binding protein 2; ALDOA: aldolase A; HK2: hexokinase 2; SORBS3: SH3 domain-containing 3; FBXO2: F-box protein 2.

Cirrhosis, representing the terminal phase of numerous chronic liver disorders, has experienced a notable global rise in incidence, escalating from 20.7 cases per 100,000 individuals in 2000 to 23.4 cases per 100,000 individuals in 2015, reflecting a 13% increase (Refs 53, 54). In response to permanent hepatic injuries, quiescent hepatic stellate cells (HSCs) undergo activation and trans-differentiate into myofibroblasts with α-smooth muscle actin positivity, which, are the primary contributors to collagen synthesis in the process of fibrosis (Refs 55, 56). A significant relationship exists between histone lactylation and the pathogenesis of liver fibrosis. Wu et al. established both in vivo CCl₄-induced fibrosis models in Sprague–Dawley rats and in vitro models using the LX-2 human hepatic stellate cell line, and observed a pronounced upregulation of H3K18 lactylation. The knockdown of LDHA was found to inhibit H3K18 lactylation, thereby attenuating HSC activation and exerting a protective effect against fibrotic alteration (Ref. 57). Furthermore, Hyunsoo et al. isolated primary HSCs from C57BL/6 mice and demonstrated that HK2 promotes histone lactylation, enhances glycolytic activity and lactate production and activates HSCs through H3K18la, which is markedly involved in liver fibrosis (Ref. 58). In a study on exercise-induced liver fibrosis, Liu et al. analysed data from 1,255 participants selected from 24,814 individuals in the National Health and Nutrition Examination Survey cohort and, together with studies using Myf5-Cre, LDHA^fl/fl^, FBXO2^fl/fl^ and SORBS3^fl/fl^ mouse models, demonstrated that excessive exercise leads to substantial lactate accumulation in skeletal muscle. This elevated lactate level stimulates the upregulation of SH3 domain-containing 3, which in turn promotes the formation of F-box protein 2 (FBXO2)-positive small secretory vesicles. Upon uptake by the liver, these small secretory vesicles transport FBXO2 to trigger the degradation of MCL1, a gene implicated in cell survival, consequently giving rise to hepatic injury (Ref. 59). Zhou et al. unveiled that the m6A-binding protein insulin-like growth factor 2 mRNA-binding protein 2 (IGF2BP2) is significantly upregulated in activated HSCs (cirrhotic, *n* = 46; healthy, *n* = 22). Furthermore, IGF2BP2 enhances lactate production and elevates H3K18la levels by modulating the key glycolytic enzyme aldolase A (ALDOA) (Ref. 60). As shown in Figure 3, increased histone H3K18 lactylation promotes transcriptional activation of profibrotic genes, thereby driving HSC activation and extracellular matrix production.

### Hepatocellular carcinoma

#### Hepatocellular carcinoma

Globally, HCC is the most prevalent form of primary liver cancer, usually occurring in the setting of chronic liver diseases such as cirrhosis (Ref. 61). In 2020, the estimated global incidence of HCC was between 15 and 30 per 100,000 individuals. Over 70% of cases occur in Asia, where it ranks as the fifth most common cancer and the second leading cause of cancer-related deaths (Ref. 62). The varying prevalence of HCC across different global regions reflects the influence of distinct aetiologies. Looking at East and Southeast Asia regions, chronic hepatitis B virus (HBV) infection stands as the principal risk factor (Refs 63, 64). In contrast, Western countries have seen a decline in HBV-related HCC cases thanks to broad HBV vaccination and effective antiviral therapies (Ref. 65); obesity and related metabolic dysfunction have emerged as more common precipitating factors (Ref. 66). For early-stage HCC, liver transplantation is considered the best treatment option (Ref. 67), and targeted therapeutic agents such as sorafenib and lenvatinib have been approved for HCC management (Ref. 68). However, due to the complex pathogenesis of HCC, it is imperative to explore novel therapeutic strategies.

Using lactylome analysis techniques, Hong et al. examined the expression profiles of lactylated proteins in normal liver tissues (*n* = 3), primary HCC tissues (*n* = 3), and HCC tissues with lung metastasis (*n* = 3) (Ref. 69). They revealed that substantial lactylation occurs in both liver and HCC tissues, with varying abundance levels across different groups. Specifically, they validated the lactylation levels of two tumour-associated proteins, ATP-Binding Cassette family 1 (ABCF1) K430la and Ubiquitin specific peptidase 14 K336la, and proposed them as specific indicators for HCC. Significant lactate accumulation inside cells is a result of the Warburg effect in HCC. In this regard, Yang et al. performed integrated lactylome and proteome analyses on an HCC cohort (*n* = 110), ultimately identifying 9,275 lysine lactylation (Kla) sites (Ref. 70). Notably, 9,256 of these Kla sites were located on non-histone proteins. Their study further demonstrated that higher levels of AK2 Kla in tumour tissues correlate with poorer prognosis. Consequently, they concluded that Kla modification contributes to HCC progression by promoting abnormal metabolic processes.

Golgi phosphoprotein 73 (GP73), highly expressed in HCC cells, serves as a crucial biomarker for diagnosing and evaluating disease progression (Ref. 71). Research by Ye et al. unravelled that GP73 functions as a key gene promoting angiogenesis in the context of HCC (Ref. 72). GP73 expression is promoted by H3K18la, while genetic deficiency of p300 reduces the expression of both H3K18la and GP73. It has been proposed that p300, acting as a ‘writer’, facilitates H3K18la expression, subsequently activating GP73 and enhancing neovascularisation via the JAK/STAT3 pathway. In a word, GP73 is transcriptionally upregulated through increased H3K18 lactylation at its promoter, thereby linking metabolic lactate accumulation to enhanced GP73-mediated angiogenesis. This indicates that GP73 acts as a downstream effector of lactylation-driven epigenetic remodelling. Another critical factor in HCC is endothelial cell-specific molecule 1 (ESM1), a secreted proteoglycan that is overexpressed in HCC to accelerate tumour growth, invasion and migration (Ref. 73). Zhao et al. found that H3K9la and H3K56la promote HCC cell proliferation, migration, invasion and the epithelial–mesenchymal transition process by binding to the ESM1 promoter and activating its transcription. This coincides with the aberrant increase in lactylation in HCC and its association with a poor prognosis (Ref. 74). Mechanistically, histone lactylation – particularly H3K9la and H3K56la – directly increases ESM1 promoter accessibility and transcription. Thus, the oncogenic functions of ESM1 in HCC are driven by lactylation-dependent epigenetic activation rather than ESM1 expression alone. Centromere protein A (CENPA) plays a vital role in centromere integrity and function during mitosis; dysregulation of CENPA leads to aneuploidy during cell division, partially responsible for tumourigenesis (Ref. 75). Research by Liao and colleagues has indicated that CENPA levels are significantly higher in HCC tissues and are related to poor prognosis (Ref. 76). HCC growth is suppressed by CENPA knockdown in light of in vitro and in vivo experiments. Its mechanism is attributable to lactylation at the K124 site of CENPA with increased transcriptional activation. Lactylated CENPA enhances its affinity to transcription factor YY1, whose binding forms a transcriptional complex to upregulate the gene expression associated with metastasis, like Cyclin D1 and Neuropilin-2. Targeting the CENPA/YY1 axis may provide a therapeutic strategy for HCC. In brief, CENPA undergoes site-specific lactylation at K124, which strengthens its interaction with YY1 and enhances transcriptional activation of downstream oncogenic targets. This demonstrates that CENPA-K124 lactylation is not merely associated with cancer progression but is functionally required for its pro-tumourigenic activity. Jin et al. investigated the role of Cyclin E2 (CCNE2) in HCC (Ref. 77). CCNE2 is essential for HCC development and linked to tumour growth and prognosis. Their findings implicated that CCNE2 correlates with HCC proliferation, migration and invasion. Importantly, CCNE2 is lactylated at lysine 348, which enhances its stability and promotes cell-cycle progression. Loss of SIRT3 increases CCNE2-K348 lactylation, indicating that lactylation is a critical PTM driving the oncogenic function of CCNE2 in HCC.

Within the tumour microenvironment (TME), a complex ecosystem comprising diverse immune cells, tumour-associated fibroblasts, endothelial cells, and the extracellular matrix, among other components, exists (Ref. 78). The accumulation of lactate in the TME can lead to extracellular acidification, which impairs the function of T cells and NK cells alongside enhancing the immunosuppressive functions of tumour-associated macrophages, myeloid-derived suppressor cells, and regulatory T cells (Tregs), jointly accelerating malignancy progression. Additionally, lactate facilitates hypoxia and angiogenesis, further amplifying the immunosuppressive impact of the TME (Ref. 17). Cai et al. discovered that upon lactate stimulation, macrophages exhibit significantly enhanced expression of nuclear protein 1(NUPR1) (a factor overexpressed in tumour cells), ultimately fostering an immunosuppressive TME. This effect has proved to be mediated through the promotion of lactylation modification (Ref. 79). Within the TME, lactate influences the TME and augments tumourigenesis by modulating the lactylation of the membrane-organising extension spike (MOESIN) protein in Treg cells to boost TGF-β signalling (Ref. 80).

Liver cancer stem cells (LCSCs) represent a cell population capable of self-renewal and differentiation into tumour cells. They drive HCC development and recurrence, and reside within malignant tissues (Ref. 81). Accumulating evidence denotes that LCSCs constitute a small subpopulation within liver tumours endowed with self-renewal, differentiation and tumourigenic attributes. They initiate HCC occurrence and influence its proliferation, invasion, metastasis, recurrence and drug resistance (Ref. 82). It is suggested that glycolytic levels are significantly higher in LCSCs in comparison with neighbouring HCC cells (Ref. 83). This elevated glycolytic flux results in increased intracellular lactate accumulation, thereby causing substantially higher levels of lactylation synchronously. High lactate levels lead to increased histone H3K56la and ALDOA K230/322 lactylation in LCSCs, which subsequently initiates the transcription of stemness-associated genes. Furthermore, LDHA inhibitors downregulate lactylation modifications at histone H3K56 and ALDOA K230/322, reducing the tumourigenic capacity of LCSCs.

**Figure 4.**
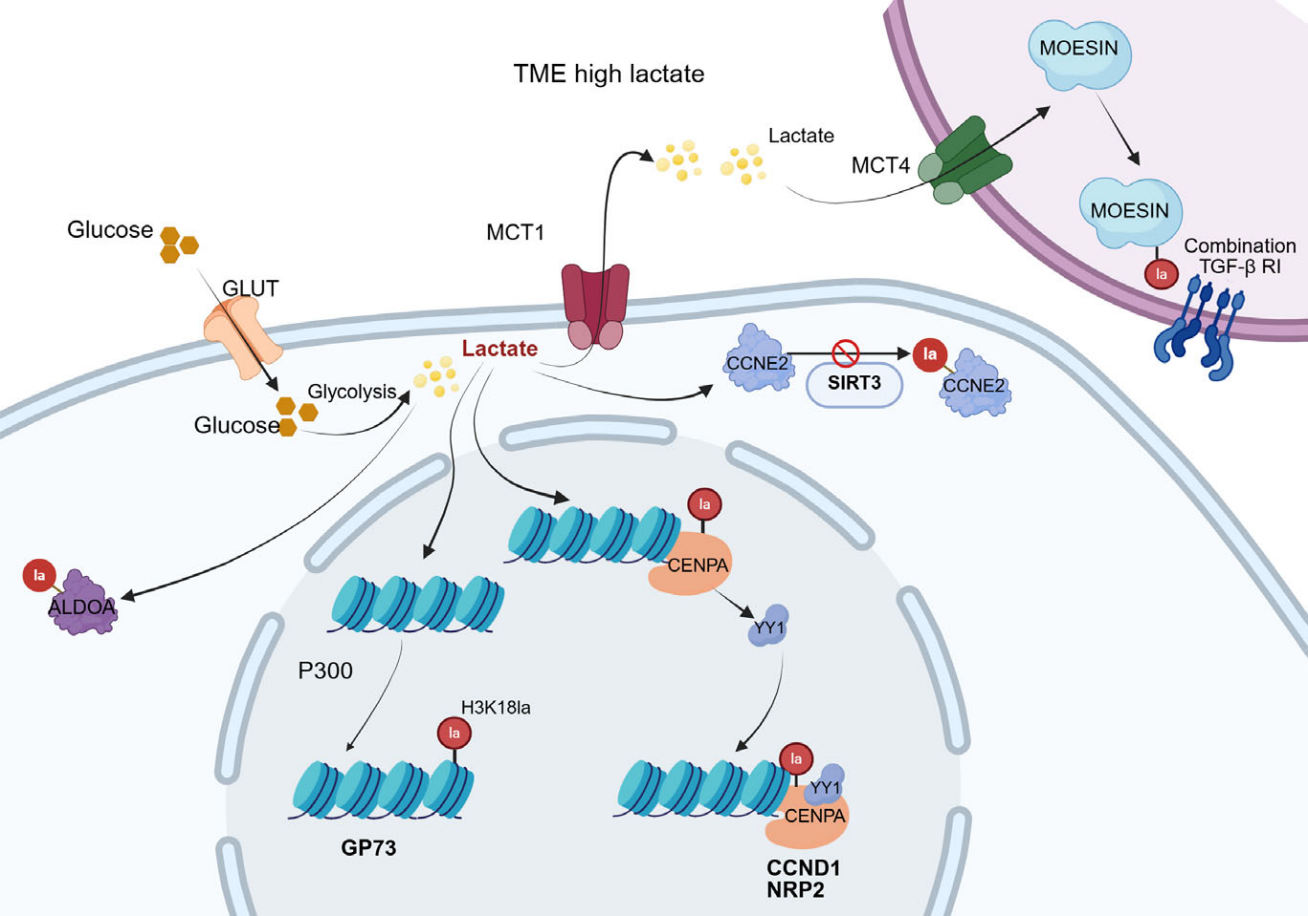
The Warburg effect drives intracellular lactate accumulation in HCC. Lactate-mediated lactylation of H3K18la promotes transcription of the GP73 gene. Reduced SIRT3 levels result in elevated lactylation of CCNE2, which stabilizes the CCNE2 protein and thereby facilitates HCC proliferation. Furthermore, lactylation of CENPA enhances expression of the transcription factor YY1. This leads to binding between lactylated CENPA and YY1, forming a transcriptional complex that amplifies the expression of genes associated with tumour metastasis. Within the TME, accumulated lactate is transported into Tregs via MCTs, promoting lactylation of MOESIN. Lactylated MOESIN, by binding to TGF-β RI, potentiates downstream SMAD3 signalling, ultimately contributing to tumour immune escape. HCC: hepatocellular carcinoma; GP73: golgi phosphoprotein 73; SIRT3: sirtuin 3; CCNE2: Cyclin E2; CENPA: centromere protein A; TME: tumour microenvironment; MOESIN: membrane-organising extension spike; SMAD3: mothers against decapentaplegic homolog 3.

Beyond histone lactylation, lactate produced by HCC also induces lactylation modifications on non-histone proteins. As previously mentioned in this review regarding the non-histone protein ABCF1, Hong et al. further discovered that lactylation at its K430 site activates the hypoxia-inducible factor 1(HIF1) signalling pathway. HIF1A, a key factor in this pathway, promotes glycolysis and lactate production by inducing LDHA expression and repressing PGC-1β expression. This ultimately establishes a lactate-ABCF1(430Kla)-HIF1A-lactate closed-loop feedback circuit (Ref. 84). Notably, p300 has been addressed as the ‘Writer’ catalysing lactylation of ABCF1, while HDAC1 and HDAC3 function as delactylases, that is, ‘Erasers’. ABCF1 is modified by lactylation at lysine 430, which facilitates activation of the HIF-1α transcriptional programme. The K430la modification is required for ABCF1’s ability to enhance malignant phenotypes, establishing lactylation as a functional driver rather than a passive marker. The findings as a whole highlight the critical role of lactylation in HCC, which is of considerable importance for diagnosis, treatment and prognosis. A broader overview of lactylation-dependent mechanisms in HCC is presented in Figure 4.

In HCC intervention and treatment, a model of lenvatinib resistance is employed to discover that lactylation preserves the insulin-like growth factor 2 mRNA-binding protein 3 (IGF2BP3) – phosphoenolpyruvate carboxykinase 2 (PCK2) – S-adenosylmethionine (SAM)- N6-methyladenosine (m6A) pathway, ensuring the expression levels of NRF2 alongside PCK2 remain constant. This improves the antioxidant capability and supports lenvatinib resistance. Conversely, lipid nanoparticles carrying IGF2BP3-targeting siRNA or the glycolytic inhibitor 2-deoxy-D-glucose (2-DG) could restore in vivo sensitivity to lenvatinib (Ref. 85). Royal jelly acid (RJA) can suppress HCC tumourigenicity by modulating downstream molecular targets of lactate and inhibiting histone H3 lactylation modification at the H3K9la and H3K14la sites (Ref. 86). This further supports the critical role of reducing histone H3 Kla levels to combat HCC and highlights the potential therapeutic value of RJA for HCC treatment.

#### Intrahepatic cholangiocarcinoma

Based on anatomical origin, cholangiocarcinoma (CCA) is classified into intrahepatic cholangiocarcinoma (iCCA) and extrahepatic cholangiocarcinoma (eCCA). In Western countries, CCA is less common than HCC. Currently, the incidence of intrahepatic CCA is rising in the United States and the United Kingdom. Increased CCA incidence is primarily ascribed to the rising prevalence of obesity and metabolic derangement across Western nations (Refs 87, 88). Conversely, in Southeast Asian countries, CCA is associated with regional factors, such as liver fluke infection (Ref. 89). CCA exhibits chemotherapy resistance and a dire outcome, resulting in a 5-year survival probability of below 20% (Refs 90, 91).

In the nucleolus, nucleolin (NCL) is the most abundant RNA-binding protein and is involved in RNA binding and splicing. Studies have confirmed its close link with tumourigenesis (Ref. 92). Through lactylome analysis, Yang et al. identified that NCL undergoes lactylation at lysine 477 (K477la) catalysed by p300. This lactylation event upregulates the expression level of MAP kinase-activating death domain protein (MADD) through an RNA splicing-dependent mechanism to activate the MAPK signalling pathway and exacerbate iCCA progression (Ref. 93).

#### Metastatic liver cancer

The primary site for colorectal cancer (CRC) metastasis is colorectal cancer liver metastasis (CRLM), which also significantly contributes to patient mortality. Studies indicate that 15%–25% of CRC subjects have synchronous liver metastases at initial diagnosis, while an additional 20%–30% develop metachronous liver metastases following primary tumour resection. The 5-year survival rate for patients with CRLM is considerably less than for those with early-stage CRC who do not have metastasis (Ref. 94).

Current research concerning the specific mechanisms of lactylation modification in CRLM encompasses multiple aspects, including the role of lactate as a signalling molecule in TME and the impact of lactylation on the function of both tumour cells and stromal cells. The gut microbiota in CRLM patients promotes intratumoural glycolysis alongside lactate production. This lactate induces lactylation modification at lysine 853 (K853la) of retinoic acid-inducible gene I (RIG-I) in macrophages, which reduces MAVS expression and subsequent nuclear factor kappa B (NF-κB) activation. Consequently, this induces M2-like polarisation of macrophages, negatively affecting immunosurveillance and promoting tumour progression (Ref. 95). The p53 protein, a critical tumour suppressor, prevents malignant cell transformation by activating numerous target genes. Its function is frequently suppressed in tumours, and its activity is also modulated in the manner of PTMs (Ref. 96). Zong et al. found that lactate derived from tumours is capable of inhibiting p53 function (Ref. 97). Through a genome-wide CRISPR screen, alanyl-tRNA synthetase 1 (AARS1), which catalyses the attachment of alanine to tRNA, has been identified as the protein in the strongest correlation with lactylation modification. Accordingly, AARS1 catalyses lactylation at lysine 120 (K120la) and lysine 139 (K139la), inactivating p53 and promoting tumourigenesis. Intriguingly, exercise supplement β-alanine competes with lactate for binding to AARS1, thereby enhancing the efficacy of cancer chemotherapy.

The G protein-coupled receptor (GPCR) family, along with G proteins and their downstream signalling molecules, plays a pivotal role in tumour initiation and development. On one hand, GPCRs influence abnormal cell proliferation and survival by activating MAPKs, AKT/mTOR and Hippo signalling pathways. On the contrary, GPCRs can encourage tumour invasion and metastasis by altering the cytoskeleton and activating Rho GTPases (Refs 98, 99). In CRLM, GPR37, a member of the GPCR family, exhibits high expression and is associated with an increase in both the size and number of liver metastatic lesions (Ref. 100). Furthermore, animal experiments have shown that knocking down or inhibiting GPR37 could ameliorate CRLM. By activating the Hippo pathway, GPR37 promotes glycolysis, a process that produces lactate. This lactate subsequently increases histone H3 lysine 18 lactylation (H3K18la), leading to higher expression levels of CXCL1 and CXCL5. As a result of this upregulation, neutrophils gather within the CRLM microenvironment.

### Acute liver injury

Acute liver injury (ALI) is caused by issues including but not limited to incorrect drug intake, alcoholism, virus and ischaemia–reperfusion (Ref. 101). Characterised by liver cell degeneration and necrosis, it leads to a rapid decline in liver function (Ref. 102). In serious cases, it can advance to liver failure or death, with liver transplantation being the only effective solution (Refs 103, 104). The aetiologies pertinent to ALI are heterogeneous; however, they often converge on several key pathological processes, including the burst of oxidative stress, activation of inflammatory cascades and disruption of energy metabolism. In recent years, the potential role of protein lactylation modification in these interactive pathological pathways has garnered burgeoning interest. This review will subsequently explore the putative roles and underlying mechanisms of lactylation modification in several representative forms of ALI.

#### Acetaminophen-induced acute liver injury

Acetaminophen (APAP) overdose is a leading cause of acute liver failure and represents the most widely used agent responsible for intrinsic drug-induced liver injury (Ref. 105). Although relatively safe, the extensive use of APAP, particularly during the COVID-19 pandemic, has resulted in increased misuse reports ascribed to a persistent elevation of APAP-triggered acute hepatic damage and subsequent organ failure (Ref. 105). The hepatotoxic mechanism involves the metabolic conversion of APAP into overwhelming amounts of the reactive electrophile N-acetyl-p-benzoquinone imine, which depletes hepatic glutathione (GSH). This depletion triggers mitochondrial dysfunction, an augmentation of reactive oxygen species (ROS), and DNA damage, partially responsible for necrotic hepatocyte death (Refs 106, 107). When exploring the impact of peroxisome proliferator-activated receptor gamma coactivator 1-alpha (PGC-1α) on liver injury owing to APAP, Hong et al. demonstrated that the activation of the SIRT1/PGC-1α/LDHB axis promotes lactate conversion to pyruvate, thereby decreasing lactylation modification in liver cells and reducing APAP-induced liver injury. The upregulation of PGC-1α is at the centre of this hepatoprotective effect (Ref. 108). In the microenvironment of APAP-induced ALI, lactate exacerbates hepatic damage by upregulating Caspase-11 expression and enhancing gasdermin D (GSDMD) activation, thereby accelerating macrophage pyroptosis (Ref. 109). Mechanistic studies revealed that lactate triggers lactylation modification at the K33 site of neural precursor cell expressed, developmentally down-regulated 4 (NEDD4) to surpass the protein–protein interaction between Caspase-11 and NEDD4. This inhibitory effect attenuates the ubiquitination of Caspase-11 by NEDD4. Furthermore, inhibition of lactylation modification was demonstrated to effectively reduce non-canonical pyroptosis in macrophages, consequently alleviating liver injury.

#### Ischaemia/reperfusion-induced liver injury

Hepatic ischaemia/reperfusion (I/R) injury is a major complication of liver resection, liver transplantation, and conditions such as abdominal trauma, haemorrhagic shock and myocardial ischaemia (Ref. 110). I/R injury is a prominent risk factor for graft rejection in the context of liver transplantation (Ref. 111). Therefore, mitigating I/R injury holds substantial clinical significance, as it can improve patient outcomes and protect the life-saving donor liver pool.

I/R injury comprises an early phase of ischemic damage to hepatocytes and a later phase characterised by the activation of immune cells and initiation of inflammatory cascades triggered by damage-associated molecular patterns (DAMPs) released upon reperfusion. HMGB1, primarily localised in the nucleus under physiological conditions, serves as a representative DAMP. When actively secreted or passively released from necrotic cells into the extracellular space, HMGB1 recruits and activates macrophages to elicit inflammatory activities (Refs 111, 112). HMGB1 undergoes lactylation modification within macrophages and is subsequently secreted extracellularly via exosomes (Ref. 113). Heat shock protein A12A (HSPA12A) has been documented to modulate glycolytic activity and influence lactate production (Ref. 114). Du et al. found that following I/R, macrophage chemotaxis is enhanced while hepatocellular HSPA12A expression is reduced. Overexpression of HSPA12A significantly mitigates I/R-induced macrophage chemotaxis, liver dysfunction and mortality (Ref. 115). The study proposed that HSPA12A disrupts the inflammatory-cytotoxic cycle between hepatocyte injury and macrophage chemotaxis/activation under I/R conditions. The underlying mechanism involves HSPA12A reducing lactate production by inhibiting glycolysis (via downregulating hexokinase 2 (HK2), LDHA, alongside pyruvate kinase M2 (PKM2) gene expression). This reduction in lactate subsequently decreases the level of lysine lactylation on HMGB1 and diminishes its paracrine secretion (primarily via exosomes). Importantly, the protective effect of HSPA12A in reducing HMGB1 paracrine secretion could be recapitulated by either reducing lactylation levels using the p300 inhibitor C646 or inhibiting glycolysis using sodium oxamate. Another research investigating I/R injury occurring during liver transplantation examined patients stratified into a hyperlactatemia group (≥ 2.1 mmol·L^−1^, *n* = 18) and a control group (< 2.1 mmol·L^−1^, *n* = 12), and proposed that lactate accumulation during the surgical procedure enhances lactylation at the K100 site of phosphoenolpyruvate carboxykinase 2 (PCK2), a key gluconeogenic enzyme regulated by KAT8 (Ref. 116). This lactylation event inhibits the polyubiquitination of oxidosqualene cyclase, leading to metabolic remodeling characterised by boosted mitochondrial fatty acid synthesis, oxidative phosphorylation (OXPHOS) and TCA cycle activity. Furthermore, targeted inhibition of PCK2 significantly ameliorates hyperlactatemia-mediated ferroptosis during hepatic I/R.

### Viral hepatitis

HBV infection represents a major global health burden. HBV infection can cause both acute and chronic liver diseases. Acute infection may progress to acute liver failure, where HBV-associated acute liver failure (HBV-ALF) carries an extremely poor short-term prognosis, with a 28-day mortality rate as high as 40%–50% (Ref. 117). Furthermore, approximately one million deaths annually are attributed to liver failure caused by HBV infection. Chronic infection predominantly leads to liver cirrhosis and HCC. Consequently, HBV infection remains a critical public health issue. Current research exploring the relationship between HBV infection and lactylation modification is relatively limited.

Zhou et al. discovered that HBV activates nicotinamide phosphoribosyltransferase (NAMPT) via the DNA damage sensor ATM and Rad3 related (ATR) (Ref. 118). This NAMPT-dependent HSC crosstalk, mediated through the insulin receptor (INSR), promotes the activation of HSCs into a myofibroblast phenotype. Moreover, HBV induces multi-site lactylation modification on poly (ADP-ribose) polymerase 1 (PARP1), leading to the activation of telomere maintenance mechanisms. Critically, experimental evidence confirmed that inhibiting the ATR-NAMPT-INSR-PARP1 axis effectively blocked HBV-induced liver fibrosis and HCC progression. Targeting this pathway may represent a promising strategy for managing chronic HBV infection. Notably, Pei et al., through bioinformatic analysis and experimental validation, identified two key lactylation-related gene biomarkers, phosphatidylinositol glycan anchor biosynthesis class M (PIGM) and class A (PIGA), in HBV-induced ALI (Ref. 119). Intriguingly, they found that methylphenidate hydrochloride, a central nervous system stimulant used to treat conditions like attention-deficit hyperactivity disorder, exhibits potential inhibitory effects on PIGM. This finding suggests a novel therapeutic avenue for repurposing existing drugs to treat HBV-ALF. In contrast, Saeed et al. analysed lactylation-related gene biomarkers specifically in HBV/HCV-associated HCC (Ref. 120). They determined six lactylation-related genes (ALB, G6PD, HMGA1, MKI67, RACGAP1 and RFC4) as potential independent prognostic biomarkers for HCC. Among these, MKI67 and RACGAP1 are specifically recognised as biomarkers with predictive value for HBV- or hepatitis C virus (HCV)–associated HCC. Validation in HCC patient samples (*n* = 60; 20 virus-negative HCC, 20 HBV-HCC and 20 HCV-HCC) demonstrated that MKI67 and RACGAP1 were significantly overexpressed in HBV/HCV-positive HCC tissues compared with virus-negative HCC.

To enhance the structural clarity of this review, the major lactylation-mediated molecules and their associated biological processes in different liver diseases are summarised in Table 2.

**Table 2.** Major lactylation-mediated biological processes and associated molecules in liver diseases

Liver disease type	Key lactylated molecules	Principal biological mechanism	Pathological effect or functional outcome	References
MAFLD (metabolic-associated fatty liver disease)	FASN, HK2, Histone H3K18la	MPC1-mediated FASN lactylation reduces hepatic lipid accumulation; HK2/H3K18la upregulation promotes glycolytic gene expression and M1 macrophage polarisation in the liver via enhanced lactylation	Exacerbates metabolic dysfunction and inflammatory activation in hepatocytes and macrophages	(Refs 51, 52)
Liver fibrosis/cirrhosis	Histone H3K18la	H3K18la enhances the transcription of profibrotic genes and facilitates hepatic stellate cell (HSC) activation	Promotes collagen deposition and fibrotic progression	(Refs 55, 56)
Acute liver injury (acetaminophen-induced)	NEDD4 (lactylated), Caspase–11	Lactylation of NEDD4 disrupts its interaction with Caspase–11, triggering non-canonical pyroptosis	Aggravates hepatocellular necrosis and tissue injury	(Ref. 109)
Ischaemia/reperfusion (I/R) injury	HMGB1, HSPA12A, PCK2 (K100la), KAT8	Lactylated HMGB1 promotes macrophage recruitment and inflammatory signaling; HSPA12A suppresses glycolysis and lactylation; KAT8-mediated PCK2 lactylation enhances mitochondrial metabolism and ferroptosis	Contributes to inflammation and cell death during hepatic I/R injury	(Refs 115, 116)
Hepatocellular carcinoma (HCC)	ESM1, NUPR1, MOESIN, ABCF1 (K430la), ALDOA (K230/322la), Cyclin E2, H3K9la, H3K14la, H3K18la, H3K56la	Histone and non-histone lactylation regulate multiple signaling pathways, promoting angiogenesis, glycolysis, immune suppression, and maintenance of stemness; SIRT3 functions as a delactylase counteracting tumour progression	Facilitates HCC cell proliferation, invasion, metastasis, and drug resistance	(Refs 70, 74, 77, 79–86, 93)
Intrahepatic cholangiocarcinoma (iCCA)	Nucleolin (NCL, K477la)	Lactylation of NCL upregulates MADD expression through alternative RNA splicing, activating the MAPK pathway	Promotes iCCA cell proliferation and tumour progression	(Ref. 93)
Colorectal cancer liver metastasis (CRLM)	RIG-I (K853la), AARS1 (K120la/K139la)	Lactylated RIG-I drives M2 macrophage polarisation; AARS1 lactylation leads to p53 inactivation	Promotes immune evasion and hepatic metastasis	(Refs 95–97)
HBV-related liver disease	PARP1 (multi-site lactylation), NAMPT, INSR, PIGM, PIGA, RACGAP1, MKI67	HBV activates the ATR–NAMPT–INSR–PARP1 axis, inducing PARP1 lactylation; multiple lactylation-associated genes serve as potential biomarkers for HBV/HCV-related HCC and acute liver failure	Drives liver fibrosis, HCC progression, and acute hepatic failure	(Refs 118–120)

## Targeted therapies for lactylation

Lactylation modification, particularly histone lactylation, serves as a pivotal link connecting cellular metabolic status with epigenetic reprogramming. It is unequivocally argued to be the core in the pathogenesis of multiple liver diseases. By modulating inflammatory responses, fibrotic processes, cellular stress responses and the expression of genes associated with malignant transformation, lactylation actively drives disease progression. Given its dramatically pathogenic contributions to chronic liver injury, liver fibrosis, and HCC, targeting the generation, recognition or function of lactylation modification has emerged as a highly promising and novel therapeutic strategy to intervene in the progression of liver diseases. Table 3 summarizes therapeutic agents documented in the literature that target lactylation for the treatment of liver diseases.

**Table 3.** Targeting lactate metabolism and lactylation in liver disease therapeutics

Drug	Target	Mechanism	References
DML	LDHA	Inhibits LDHA, reducing lactylation levels at H3K9 and H3K56, suppresses proliferation and migration of LCSCs and induces apoptosis.	(Ref. 125)
Oxamate	LDHA	Reduces the size and stem cell frequency of tumour spheroids formed by LCSCs	(Ref. 83)
GSK2837808A	LDHA	Enhances the anti-tumour efficacy of anti-PD–1 therapy and delays HCC progression	(Ref. 80)
Salvianolic acid B	LDHA	Downregulates LDHA expression, inhibiting H3K18 lactylation and alleviating hepatic injury.	(Ref. 126)
RJA	LDHA	Inhibits LDHA, preventing lactylation modifications at H3K9 and H3K14.	(Ref. 86)
LOx	Lactate	Reduces lactate concentration, releasing H_2_O_2_ and recruiting immune cells, thereby overcoming immunosuppression and sensitising to immunotherapy.	(Ref. 130)
CHC	MCT1	Inhibits MCT1, preventing lactate-induced NEDD4 lactylation and suppressing atypical pyroptosis.	(Ref. 109)
BAY8002	MCT1	Inhibits MCT1, decreasing lactate levels and enhancing anti-PD–1 therapeutic efficacy in HCC.	(Ref. 133)
Syrosingopine	MCT1/4	Blocks MCT1 and MCT4 function.	(Ref. 134)
AC–73	MCT1/4	Disrupts CD147 dimerisation, suppressing the CD147/ERK1/2/STAT3/MMP–2 signalling pathway.	(Ref. 136)
CCS1477	p300	p300 inhibitor	(Ref. 137)
FT–7051	p300	p300 inhibitor	(Ref. 137)
SRT 2183	SIRT 1	SIRT1 agonist reduces NEDD4 lactylation, alleviates APAP-induced hepatic injury and delays HCC progression.	(Ref. 109)
HKL	SIRT 3	SIRT3 agonist decreases CCNE2 lactylation, attenuates APAP-induced hepatic injury and suppresses HCC progression.	(Ref. 77)
sodium lactate solution		Immunotherapy sensitisation by potentiating antitumour immunity via CM8^+^ T cells.	(Ref. 139)

DML: demethylzeylasteral; LDHA: lactate dehydrogenase A; LCSCs: liver cancer stem cells; RJA: royal jelly acid; LOx: lactate oxidase; CHC: α-cyano–4-hydroxycinnamate; MCT1/4: monocarboxylate transporter; NEDD4: neural precursor cell-expressed developmentally down-regulated gene 4; PD–1: programmed death–1; HCC: hepatocellular carcinoma; APAP: acetaminophen; SIRT 1/3: sirtuin1/3; HKL: Honokiol.

### Inhibiting lactate production

Lactate, a crucial metabolite of cellular respiration, serves as both an energy source and a signalling molecule regulating neuronal action, and the balance between pro-inflammatory and anti-inflammatory responses contextually (Refs 121–123). Given that lactate can induce lactylation modification and promote its elevation, inhibiting lactate generation therefore represents a strategy to suppress lactylation modification-associated progression of liver diseases (Refs 5, 124).

LDHA, the key enzyme responsible for lactate production during glycolysis, is a prime target for inhibiting lactate generation. Numerous studies have focused on LDHA and its effect in mediating lactylation. Pan et al. found that demethylzeylasteral (DML), a triterpenoid compound isolated from Tripterygium wilfordii, exhibits anti-tumour activity in LCSCs (Ref. 125). DML inhibits LDHA, thereby reducing lactate levels and significantly decreasing lactylation modification at the H3K9 and H3K56 sites. Consequently, DML suppresses LCSC proliferation and migration while promoting LCSC apoptosis. Feng et al., investigating lactylation at the K230/K322 sites of aldolase A (ALDOA) in LCSCs, demonstrated that the LDHA inhibitor sodium oxamate (Oxamate) significantly inhibited the size and stem cell frequency of tumour spheroids formed by LCSCs, and reduced the mRNA and protein expression of LCSC stemness markers (Ref. 83). Gu et al. utilised the LDHA inhibitor GSK2837808A in a mouse model of HCC, showing that it strengthens the anti-tumour efficacy of anti-PD-1 therapy and delays HCC progression (Ref. 80). Hu et al. applied salvianolic acid B (Sal B), an extract from *Salvia miltiorrhiza*, in a hepatic damage model challenged by CCl_4_ administration (Ref. 126). They found that Sal B downregulates LDHA expression, exerts an inhibitory effect on lactylation at the H3K18 site, and consequently mitigates liver injury. Xu et al. discovered that RJA inhibits HCC development by suppressing LDHA and thereby preventing lactylation modification at the H3K9 and H3K14 sites (Ref. 86). This study has not only confirmed the beneficial effect of LDHA inhibition in ameliorating HCC but also elaborated on the critical role of reducing histone H3 Kla levels in combating HCC.

Targeting lactate dehydrogenase (LDH) to reduce lactate production has demonstrated considerable potential, as evidenced by numerous studies. However, concerns persist regarding its metabolic disruption towards normal cells as well as non-specific toxic effects. As a potential approach to overcome the aforesaid limitations, lactate oxidase (LOx) presents an alternative therapeutic option. LOx reduces lactate levels, releases H₂O₂ and recruits immunocytes, thereby mitigating immunosuppression and sensitising tumours to immunotherapy (Ref. 127). Following suboptimal thermal ablation therapy, residual tumours exhibit a more pronounced immunosuppressive state, which accelerates disease progression and contributes to therapy resistance. Chen et al. found that increased intratumoural lactate accumulation, driven by enhanced glycolytic activity in residual HCC cells post-ablation, is capable of exacerbating the immunosuppressive TME (Ref. 128). Administration of LOx continuously depletes intratumoural lactate through cascade enzymatic reactions. This converts the immunosuppressive TME into an immunostimulatory pattern in HCC-bearing mouse models and synergizes with immune checkpoint inhibitor therapy, significantly suppressing residual HCC growth and lung metastasis, consequently prolonging the survival of post-operative mice. Conversely, HCC treatment might be improved by inducing ferroptosis, a regulated form of cell death, although elevated levels of histone lactylation contribute to tumour resistance against ferroptosis (Ref. 129). Chen et al. developed and applied nanoparticles loaded with LOx (Ref. 130). By leveraging the high lactate levels in tumour tissue to generate substantial H₂O₂, these nanoparticles induce ferroptosis in HCC. The accumulation of H₂O₂, catalysed by LOx, significantly elevates intracellular ROS levels to potently trigger ferroptosis.

### Inhibiting lactate transport

Lactate is not only produced within cells but can also be transferred between them via MCTs, which assist in its transport. Targeting MCTs can therefore disrupt lactate release (Ref. 131). Lactate is primarily transported by MCT1, MCT2 and MCT4. MCT1 and MCT2 are responsible for bringing lactate into the cell from the extracellular environment, while MCT4 helps move lactate out of the cell (Ref. 132).

In a study concerning ALI upon APAP exposure, Li et al. unveiled that inhibiting MCT1 with α-cyano-4-hydroxycinnamate (CHC) significantly halts exogenously lactate-triggered lactylation of NEDD4 in macrophages, inhibits atypical pyroptosis, and ameliorates hepatic damage (Ref. 109). Delving into the mechanism of Serine/arginine splicing factor 10 (SRSF10) – glycolysis – H3K18la in HCC tumour cells, Cai et al. showed that application of the MCT1 inhibitor BAY8002 significantly reduces extracellular lactate levels in HCC cells and enhances the potency of anti-PD-1 treatment against HCC (Ref. 133). Syrosingopine, a classic antihypertensive drug, inhibits both MCT1 and MCT4. Benjamin et al. found that Syrosingopine treatment improves hepatomegaly and reduces the number of tumour nodules in HCC-bearing mice (Ref. 134). The chaperone protein CD147 facilitates the plasma membrane expression of MCT1 and MCT4 (Ref. 135). The application of the CD147 inhibitor AC-73 primarily represses the CD147/ERK1/2/STAT3/MMP-2 pathway by disrupting CD147 dimerisation to harness HCC migration and invasion (Ref. 136).

### Inhibiting lactylation

In “Inhibiting lactate production” section, we discuss strategies that regulate lactylation by suppressing lactate production through metabolic inhibition. In contrast, the present section focuses on the direct enzymatic modulation of the lactylation modification itself. The lactylation process involves ‘writers’ and ‘erasers’, enzymes responsible for adding or removing lactate groups. Targeted interventions against these enzymes have demonstrated significant therapeutic potential in mitigating the impact of lactylation in the context of hepatopathy.

Research has focused on targeting p300 or its homolog CBP. Notably, two p300 inhibitors, CCS1477 and FT-7051, have been validated in clinical trials for hematologic malignancies (Ref. 137). Another p300 inhibitor, A485, is proven to potentiate the anti-cancer efficacy of monotherapy in combination with anti-PD-L1 antibodies in tumour-bearing animal models. SIRT1 and SIRT3 function as lactylation ‘erasers’, catalysing the removal of lactate groups. Studies reveal that SIRT1 (SRT 2183) and SIRT3 (HKL) agonists diminish the lactylation levels of respective NEDD4 and CCNE2 to alleviate liver damage from APAP and prevent the advancement of HCC (Refs 6, 138).

Curiously, the part lactate plays in tumour metabolism and immunotherapy is complicated, with some research suggesting a paradoxical notion that lactate might, under certain conditions, support tumour therapy. For instance, administering sodium lactate solution beneath the skin promotes anti-tumour immunity via CD8^+^T cells and enhances the response of tumours to immunotherapy in mouse models with CRC, non-small-cell lung cancer and melanoma (Ref. 139). Specifically, in HCC, Tian et al. leveraged the abundantly enriched lactate and mildly acidic microenvironment within liver tumour tissues to design a nanoplatform that catalyses the generation of excessive ROS from intratumoural lactate for catalytic tumour therapy (Ref. 139).

## Detection and validation of lactylation

**Figure 5.**
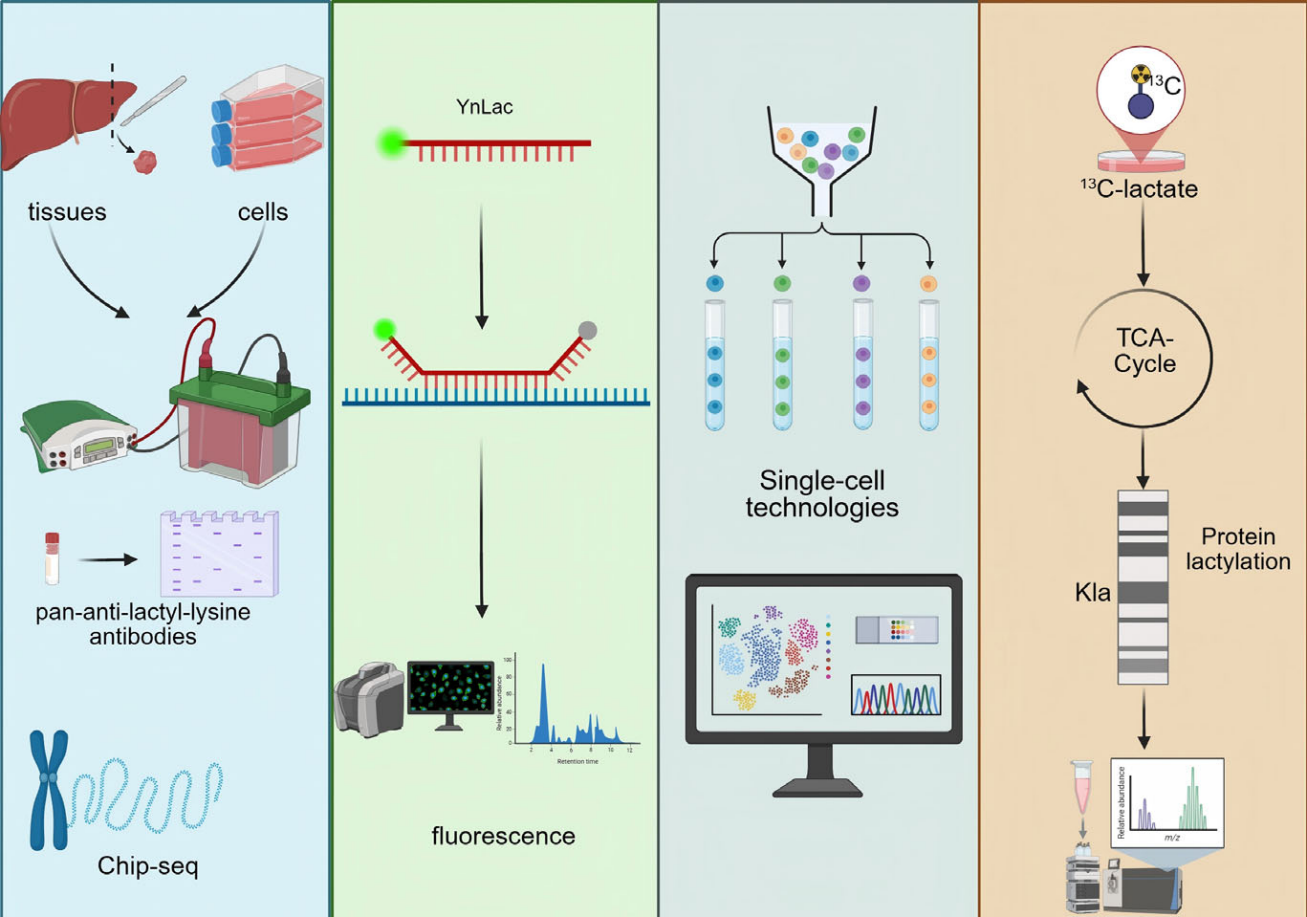
Detection and validation strategies for protein lactylation. Overview of biochemical, chemical probe-based, single-cell and isotope-tracing approaches used to detect and validate protein lactylation.

Accurate detection and rigorous validation of protein lactylation are essential for elucidating its biological functions. Multiple complementary approaches are currently employed to verify this PTM. An overview of the major detection and validation strategies for protein lactylation is schematically summarised in Figure 5. At the biochemical level, immunoblotting using pan-anti-lactyl-lysine antibodies or site-specific antibodies such as anti-H3K18la enables convenient monitoring of global or histone-specific lactylation changes. Using Western blotting, Yu et al. reported that histone Kla levels were significantly higher in ocular melanoma tissues compared with normal melanocyte samples and were positively associated with poor patient prognosis (Ref. 140). Chromatin immunoprecipitation (ChIP) assays further determine whether lactylation marks are enriched at regulatory genomic regions. For example, Cui et al. demonstrated through ChIP-qPCR and ChIP-seq that lactate-stimulated macrophages exhibit increased lactylation at the promoters of ARG1, THBS1 and VEGFA (Ref. 141).

In addition, several emerging technologies allow precise detection of lactate and lactylation, providing critical tools for dissecting their dynamic regulation. Chemical-probe-based strategies, including fluorescently labelled L-lactate analogues, enable real-time tracking of lactate transport and metabolism in living cells with high spatial and temporal resolution (Ref. 142). Sun et al. further developed the YnLac probe (sodium (S)-2-hydroxypent-4-ynoate), which can be metabolically incorporated into lactylated proteins and subsequently visualised or enriched through fluorescent or affinity tags for imaging and proteomic analysis (Ref. 143).

Single-cell technologies have also become powerful tools in lactylation research, enabling correlation of lactylation levels with gene-expression programmes at a cell-type–resolved level. Such approaches help characterize tumour metabolic phenotypes, evaluate disease prognosis, and delineate cell-level metabolic heterogeneity (Refs 144, 145). Single-cell metabolic profiling and quantitative mass spectrometry platforms can capture a spectrum of metabolites – including lactate – revealing metabolic diversity across cell populations and tumour progression, and supporting the development of lactate-based metabolic signatures for predicting metastatic risk (Refs 146, 147).

Furthermore, isotope-tracing metabolic flux analysis enables the tracking of lactate-derived carbon within intracellular metabolic pathways, helping to delineate how lactate contributes to specific metabolic branches and how these pathways connect to lactylation. Using 13C-labelled substrates, Liu et al. showed that breast cancer cell lines preferentially rely on OXPHOS during the G1 phase but shift towards glycolysis during the S phase, illustrating the dynamic metabolic context underlying lactylation regulation (Ref. 148).

Collectively, these advanced analytical strategies have substantially improved the precision of lactate and lactylation measurements, deepening our understanding of their functional roles and regulatory networks in tumour biology. A summary of these detection and validation strategies is provided in Table 4.

**Table 4.** Methods for detecting and validating protein lactylation

Technique	Description	Applications	References
Immunoblotting (pan–anti-lactyl-lysine or site-specific antibodies)	Detects global or site-specific lactylation levels via antibody recognition	Monitoring changes in histone and non-histone lactylation under different conditions	(Ref. 140)
Chromatin immunoprecipitation (ChIP-qPCR, ChIP-seq)	Enrichment of lactylated histones to assess promoter-specific lactylation	Identifying genomic loci enriched for lactylation marks	(Ref. 141)
Fluorescent lactate analogues	Real-time visualisation of lactate uptake and metabolism	Tracking lactate dynamics in live cells	(Ref. 142)
YnLac probe (sodium (S)–2-hydroxypent–4-ynoate)	Metabolic incorporation into lactylated proteins; enables fluorescent tagging or affinity enrichment	Imaging lactylated proteins; proteomic profiling	(Ref. 143)
Single-cell technologies	Correlates lactylation with transcriptional states and cell-type-specific phenotypes measures lactate and related metabolites at single-cell resolution	Tumour metabolic phenotyping; prognostic evaluation; identifying metabolic heterogeneity and lactate-driven phenotypes	(Refs 144–147)
Isotope tracing (13C-labelled lactate or glucose)	Tracks lactate-derived carbon flow through metabolic pathways	Determining metabolic routes contributing to lactylation	(Ref. 148)

## Summary

Lactate metabolic reprogramming and its derivative modification, lactylation, have emerged as a pivotal regulatory axis in the pathogenesis of liver diseases. This review systematically elucidates that aberrant lactate accumulation within the hepatic microenvironment not only directly fuels the advancement of liver fibrosis, ALF and HCC by serving as an energy substrate, driving fatty acid synthesis, and maintaining redox homeostasis but also exerts epigenetic regulatory functions through the emerging lactylation pathway. Lactylation dynamically modifies both histone and non-histone targets, profoundly participating in hepatocyte phenotypic transformation, inflammatory cascades, immune surveillance evasion and pathological angiogenesis. Consequently, it establishes a molecular bridge linking metabolic dysregulation to the malignant progression of liver diseases. Building upon this foundation, therapeutic strategies targeting the lactate pathway demonstrate significant potential: inhibiting LDH activity, blocking MCT1/4-mediated lactate shuttling or intervening with lactylation-modifying enzymes can effectively disrupt tumour metabolic symbiosis and reverse the immunosuppressive microenvironment, offering novel paradigms for liver disease treatment.

However, the field currently faces substantial challenges. Most targeted therapies remain in the early stages of clinical trials. The metabolic variation and complexity across divergent liver tumour types, clinical stages/grading and cellular populations within the TME pose significant difficulties in achieving effective drug concentrations at tumour sites. Extant inhibitors of lactylation often lack sufficient selectivity for the lactate pathway, potentially interfering with normal hepatocyte physiology and causing off-target effects. Overcoming these barriers requires multi-dimensional innovation: First, developing liver disease diagnostic models based on lactate metabolism-related gene signatures, combined with molecular imaging for dynamic monitoring of lactate metabolism. Second, designing liver-targeted nano-delivery systems to enhance drug accumulation. Many advanced drug delivery systems currently under development can efficiently ensure that therapeutics reach their deliberate sites of action at enough concentrations while minimising side effects. Finally, exploring rational and synergistic combination therapeutic strategies, such as simultaneously targeting multiple pathways, is indispensable to mitigate potential drug resistance risks and enhance overall treatment efficacy. These multifaceted approaches are essential to overcome the drawbacks of current therapies and finally achieve improved therapeutic outcomes and prognoses for patients with liver diseases.

In conclusion, research on lactate metabolism and lactylation is profoundly reshaping our understanding of liver disease mechanisms. Although clinical translation hurdles persist, integrating multi-omics analyses, innovative drug design and prospective clinical trials holds promise for developing precision intervention strategies targeting the lactate pathway. Such strategies have the potential to become a revolutionising force in improving outcomes for patients with liver diseases, ultimately shifting the paradigm of liver disease management from symptom control towards targeting the intricate interplay between metabolism and epigenetic regulation.
